# Peritumoral radiomics features on preoperative thin-slice CT images can predict the spread through air spaces of lung adenocarcinoma

**DOI:** 10.1038/s41598-022-14400-w

**Published:** 2022-06-20

**Authors:** Keiichi Takehana, Ryo Sakamoto, Koji Fujimoto, Yukinori Matsuo, Naoki Nakajima, Akihiko Yoshizawa, Toshi Menju, Mitsuhiro Nakamura, Ryo Yamada, Takashi Mizowaki, Yuji Nakamoto

**Affiliations:** 1 Department of Radiation Oncology and Image-Applied Therapy, Graduate School of Medicine, Kyoto University, 54 Shogoinkawahara-cho, Sakyo-ku, Kyoto, 606-8507 Japan; 2 Department of Diagnostic Imaging and Nuclear Medicine, Graduate School of Medicine, Kyoto University, Kyoto, Japan; 3 Department of Diagnostic Pathology, Graduate School of Medicine, Kyoto University, Kyoto, Japan; 4 Department of Thoracic Surgery, Graduate School of Medicine, Kyoto University, Kyoto, Japan; 5 Division of Medical Physics, Department of Information Technology and Medical Engineering, Human Health Sciences, Graduate School of Medicine, Kyoto University, Kyoto, Japan; 6 Department of Statistical Genetics, Graduate School of Medicine, Kyoto University, Kyoto, Japan

**Keywords:** Cancer imaging, Lung cancer

## Abstract

The spread through air spaces (STAS) is recognized as a negative prognostic factor in patients with early-stage lung adenocarcinoma. The present study aimed to develop a machine learning model for the prediction of STAS using peritumoral radiomics features extracted from preoperative CT imaging. A total of 339 patients who underwent lobectomy or limited resection for lung adenocarcinoma were included. The patients were randomly divided (3:2) into training and test cohorts. Two prediction models were created using the training cohort: a conventional model based on the tumor consolidation/tumor (C/T) ratio and a machine learning model based on peritumoral radiomics features. The areas under the curve for the two models in the testing cohort were 0.70 and 0.76, respectively (*P* = 0.045). The cumulative incidence of recurrence (CIR) was significantly higher in the STAS high-risk group when using the radiomics model than that in the low-risk group (44% vs. 4% at 5 years; *P* = 0.002) in patients who underwent limited resection in the testing cohort. In contrast, the 5-year CIR was not significantly different among patients who underwent lobectomy (17% vs. 11%; *P* = 0.469). In conclusion, the machine learning model for STAS prediction based on peritumoral radiomics features performed better than the C/T ratio model.

## Introduction

Spread through air spaces (STAS) is an invasive pattern of lung cancer that was newly described in the 2015 World Health Organization (WHO) classification^1^. STAS is defined as micropapillary clusters, solid nests, or single cells beyond the edge of the tumor into air spaces in the surrounding lung parenchyma. It is known as a negative prognostic factor in patients with early-stage lung adenocarcinoma^2,3^, especially in patients receiving limited resection^4,5^. Thus, STAS could be a potential biomarker for clinical decision-making in selecting surgical methods, such as lobectomy or limited resection, if it can be predicted preoperatively.

STAS findings are not directly visible on CT images, but there are papers that indirectly show a correlation with CT findings. Several authors suggested that tumor density is an important factor in predicting STAS, and the probability of its presence could be estimated by the consolidation tumor ratio (C/T ratio) on preoperative CT^6–9^. Kim et al. showed that among various qualitative and quantitative CT features, the percentage of solid component was an independent predictor for STAS^9^. In addition, other morphological features, such as notches, surrounding ground-glass opacity (GGO), vascular convergence, pleural indentation, and spiculation, were related to the presence of STAS^10^.

Radiomics is a quantitative approach that makes use of statistical patterns in tumor CT images to predict tumor pathology, tumor treatment response, or cancer prognosis using a large number of features extracted from medical images. Numerous studies have shown that radiomics features could quantify tumor characteristics and can potentially be applied as clinical biomarkers^11–15^. Several authors applied the radiomics approach to the prediction of STAS. Most previous studies have investigated the association between STAS and radiomics features inside the tumor^16,17^. Since STAS is a pathological finding present at the tumor edge, radiomics features at the tumor margins on preoperative CT images may lead to a more accurate prediction of STAS. Zhuo et al. had evaluated peritumoral radiomics features from preoperative CT, which resulted in no significant improvement in the prediction performance of STAS compared with a clinical model including maximal diameter of the solid component and mediastinal node metastasis^18^. This may be because the radiomics features were obtained from regions of interest (ROIs) outside the tumor contour, which may not truly represent the tumor edge characteristics associated with STAS, as described earlier, because of the limits of CT resolution. The peritumoral ring-shaped ROI, which contains both inside and outside the tumor edge, may overcome this limitation.

In the present study, we investigated the performance of machine learning models based on peritumoral radiomics features, aiming to improve the prediction performance of STAS in comparison with the conventional method using the C/T ratio.

## Methods

### Ethics

All study procedures complied with the 1964 Declaration of Helsinki and its later amendments. The study was approved by the Ethics Committee at Kyoto University Graduate School and Faculty of Medicine (approval no. R2272). As this study was performed retrospectively, the requirement for informed consent was waived.

### Patients

**Figure 1:**
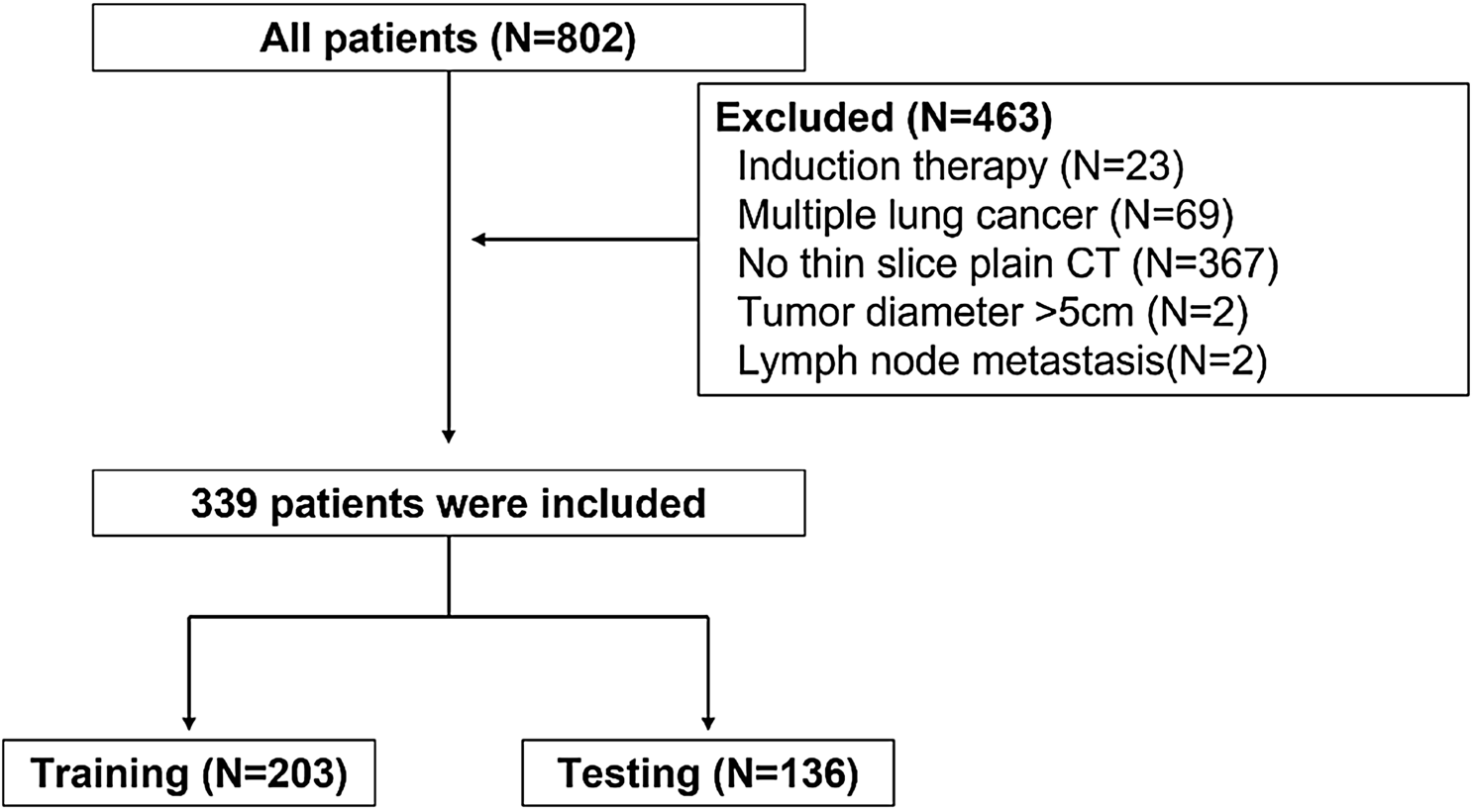
Flowchart of the inclusion/exclusion criteria.

From January 2007 to December 2015, 802 patients with pathologically confirmed lung adenocarcinoma were identified from our surgery database. Of these, 463 patients were excluded because of induction chemotherapy (n = 23), multiple lung cancer nodules (n = 69), absence of thin-slice plain CT (n = 367), tumor diameter greater than 5 cm (n = 2), and presence of lymph node metastasis (n = 2). The remaining 339 patients were included in the analyses (Fig. 1).

### Histological evaluation

Two experienced pathologists reviewed the hematoxylin and eosin tissue sections with a Nikon Eclipse 80i optical microscope (Nikon Corporation, Tokyo, Japan) according to the WHO definitions of STAS. The edge of the main tumor was defined as a smooth surface that could be easily recognized by low-power visual field examination. STAS was defined as tumor aggregates floating in the air cavity at least one alveolus away.

### Image acquisition

CT scans were performed using a 64-detector-row CT scanner (Aquillion 64, Canon Medical Systems, Otawara, Japan) or a 320-detector-row scanner (Aquillion ONE, Canon Medical Systems). Images were reconstructed with a soft-tissue kernel (FC11, 13) and a slice thickness of 1 mm for radiomics analysis and with lung kernel (FC51) and a slice thickness of 0.5 mm for evaluation of the C/T ratio, using a filtered back-projection algorithm. Table E1 enumerates the detailed scan parameters.

### Radiological evaluation of the C/T ratio and the type of nodule

The largest diameter of the whole tumor, the largest diameter of consolidation (solid part), and types of nodules (solid, part-solid, and ground-glass nodule) were determined by an experienced radiation oncologist (K.T. with ten years of experience in radiotherapy for lung cancer and in lung cancer related-image interpretation). A board-certified radiologist (R.S. with 14 years of experience in lung image interpretation) independently confirmed the results and consensus was reached by discussion in the event of disagreement. All cases were anonymized and both readers were blinded to the presence or absence of STAS and to clinical outcomes. The largest diameter of the tumor was measured on the axial, coronal, or sagittal planes of the CT in the lung window (window level, − 600 HU; window width, 1500 HU). The largest consolidation diameter was measured on the same plane where the largest tumor diameter was measured.

### Tumor segmentation and feature extraction

Peritumoral ROI was defined as a ring-shaped ROI 5 mm inward and 5 mm outward from the tumor surface, excluding surrounding soft tissues, such as the chest wall or mediastinum. A radiation oncologist (K.T.) segmented the peritumoral ROIs using 3D Slicer (version 4.10.2), which is a free, open source and multi-platform software package for medical, biomedical, and related imaging research (https://www.slicer.org/). Details on the segmentation procedures are given in Figure E1. Segmentation in randomly selected patients was also performed by a radiologist (R.S.) to assess the reproducibility of radiomics features. Dice coefficients were calculated to compare the lesion segmentation and assess the interobserver variability.

The radiomics features were extracted from peritumoral ROIs using PyRadiomics (version 3.0), supported by the image biomarker standardization initiative (IBSI)^19^. All slices were resampled to 1 × 1 mm^2^ in the horizontal and vertical directions before the feature extraction. The features included 14 shapes, 18 first-order, 22 gray level co-occurrence matrices (GLCM), 14 gray level dependence matrices (GLDM), 16 gray level size zone matrices (GLSZM), 16 gray-level run-length matrices (GLRLM), and 5 neighboring gray-tone difference matrices (NGTDM). In addition to the original image, images processed with Laplacian of Gaussian (LoG) and coiflet wavelet filters were applied for six feature classes (first-order, GLCM, GLDM, GLSZM, GLRLM, and NGTDM). Consequently, 1288 features were extracted from each ROI. A complete list of radiomics features is provided in Table E2.

### Model development

**Figure 2:**
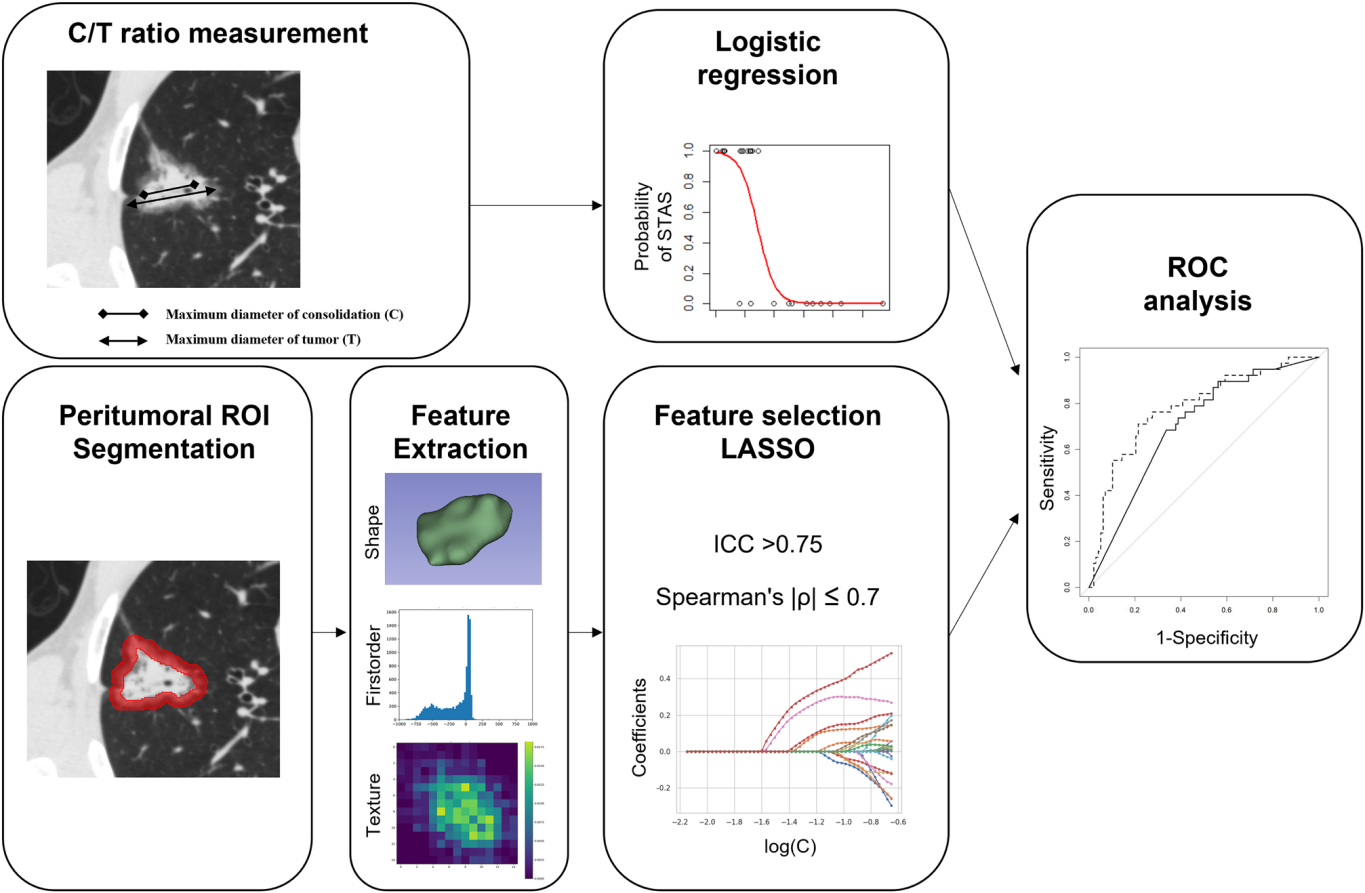
Workflow of the predictive model development.

The patient cohort was randomly divided into training and testing cohorts (3:2) using the two stratification factors (the presence of STAS and nodule types). We developed two models for the prediction of STAS (Fig. 2) in the present study. One was a machine learning model based on peritumoral radiomics features (peritumoral radiomics model). The other was a logistic regression model based on the tumor C/T ratio (C/T ratio model).

Before developing the peritumoral radiomics model, we selected non-redundant, reproducible features. The intraclass correlation coefficients (ICCs) were calculated from ROIs independently by two researchers to quantify the interobserver reproducibility of the radiomics features. An ICC of > 0.75 was considered reproducible^20^. The absolute pairwise Spearman’s correlation coefficient values were calculated to remove redundant features in the training set (|ρ| > 0.7).

Finally, the peritumoral radiomics model was developed using the least absolute shrinkage and selection operator (LASSO) classification algorithm (Python scikit-learn environment, version 0.22.1) as a peritumoral radiomics model. The regularization parameters for LASSO were tuned using fivefold cross-validation on the training dataset. The regularization parameters of the model with the highest AUC in the fivefold cross validation were used to create the final model.

### Evaluation of predictive performance

The predictive performance of the models was compared using the area under the curve (AUC) of the receiver operating characteristic curve. The 95% confidence interval (CI) for the AUC was calculated by bootstrapping with 2,000 iterations. For comparison, the *P* value was calculated by the DeLong method using the pROC package in R (version 1.16.1). The cut-off value dividing the cohort into STAS high-risk and STAS low-risk groups was selected to maximize Youden’s index for each model.

The follow-up and survival periods were calculated from the surgery day. The cumulative incidence of recurrence (CIR) was calculated using competing risk analysis, with death without recurrence regarded as a competing event. The differences in CIR between the groups were tested by the Gray’s test using the cmprsk package in R (version 2.2-9). The level of significance was set at *P* < 0.05.

## Results

### Patients

The patient cohort was divided into the training (n = 203) and testing (n = 136) cohorts. STAS was positive in 57 patients (28%) in the training cohort and 38 patients (28%) in the testing cohort. The demographic and clinical characteristics of the patients are shown in Table 1. There were no statistically significant differences in the patient characteristics between the training and testing cohorts.

**Table 1:** Patient characteristics.

Characteristics	Overall (N = 339)	Training cohort (n = 203)	Testing cohort (n = 136)	*P* value
Age	67 (61, 73)	67 (60, 74)	67 (61, 73)	0.89
**Sex**				0.61
Male	160 (47%)	93 (46%)	67 (49%)	
Female	179 (53%)	110 (54%)	69 (51%)	
**Smoking status**				0.60
Current	56 (17%)	34 (17%)	22 (16%)	
Ex	119 (35%)	67 (33%)	52 (38%)	
Never	164 (48%)	102 (50%)	62 (46%)	
**Location**				0.09
Left lower lobe	49 (14%)	22 (11%)	27 (20%)	
Left upper lobe	88 (26%)	49 (24%)	39 (29%)	
Right lower lobe	65 (19%)	43 (21%)	22 (16%)	
Right middle lobe	27 (8%)	19 (9%)	8 (6%)	
Right upper lobe	110 (32%)	70 (34%)	40 (29%)	
**T-stage**				0.96
Tis	52 (15%)	31 (15%)	21 (15%)	
T1mi	23 (7%)	14 (7%)	9 (7%)	
T1a	45 (13%)	24 (12%)	21 (15%)	
T1b	111 (33%)	70 (34%)	41 (30%)	
T1c	70 (21%)	41 (20%)	29 (21%)	
T2a	29 (9%)	18 (9%)	11 (8%)	
T2b	9 (3%)	5 (2%)	4 (3%)	
**Surgery**				0.23
Lobectomy	204 (60%)	129 (64%)	75 (55%)	
Partial resection	26 (8%)	13 (6%)	13 (10%)	
Segmentectomy	109 (32%)	61 (30%)	48 (35%)	
Diameter of consolidation (mm)	14 (6, 23)	15 (6, 22)	13 (6, 23)	0.77
Diameter of tumor (mm)	19 (15, 27)	19 (14, 27)	20 (15, 27)	0.73
C/T ratio	0.80 (0.38, 1.00)	0.81 (0.38, 1.00)	0.77 (0.38, 1.00)	0.79

Values are presented in median (interquartile range) or number (percentage).

*C/T ratio* consolidation/tumor ratio.

### Tumor segmentation, feature extraction, and model development

**Figure 3:**
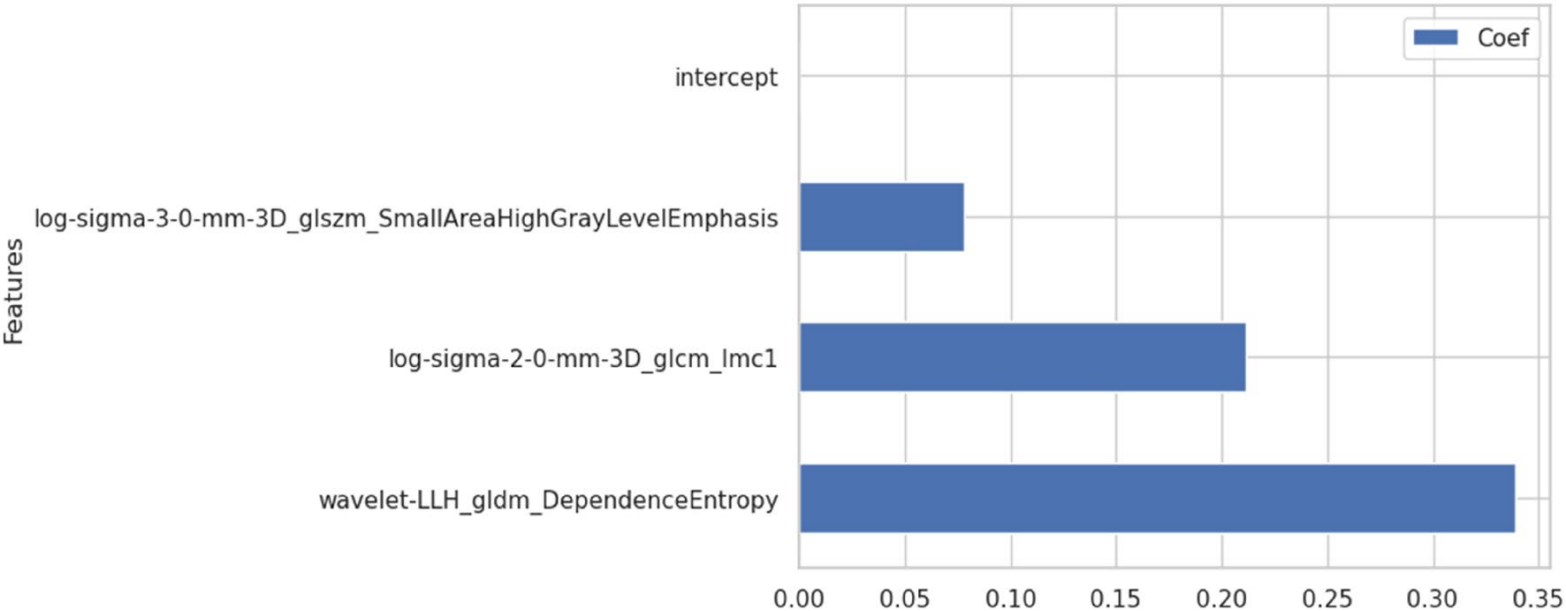
Coefficients of the peritumoral radiomics model.

The mean dice coefficient of segmentation in 102 patients (50.2%) randomly selected from the training cohort was 0.82 (range 0.47–0.99). A total of 1132 (88%) features with an ICC of ≥ 0.75 were considered to be reproducible against the interobserver variability. The ICC values for the features are shown in the supplementary material (intraclass_correlation.csv). Of these reproducible features, 88 (8%) features with an absolute value of pairwise |ρ| of ≤ 0.7 were used to develop the LASSO model. The AUCs in the fivefold cross validation of the training cohort were 0.78, 0.72, 0.71, 0.88, and 0.76 (mean, 0.77). The selected coefficients of the features in the LASSO model are shown in Fig. 3. The AUCs of the peritumoral radiomics model and the C/T ratio model in the entire training cohort were 0.79 (95% CI 0.72–0.86), and 0.72 (95% CI 0.65–0.78, Table 2), respectively. The cut-off values between the STAS high- and low-risk groups were determined to be 0.53 and 0.54 for the peritumoral radiomics and the C/T ratio models, respectively.

**Table 2:** Predictive performance of consolidation/tumor model and peritumoral radiomics model.

Patient cohort	Model	AUC (95%CI)	Sensitivity	Specificity
Training	C/T ratio model	0.72 (0.65–0.78)	0.82	0.62
	Peritumoral radiomics model	0.79 (0.72–0.86)	0.82	0.69
Testing	C/T ratio model	0.70 (0.61–0.78)	0.71	0.61
	Peritumoral radiomics model	0.76 (0.67–0.84)	0.74	0.66

*C/T ratio* consolidation/tumor ratio, *AUC* area under the curve, *CI* confidence interval.

### Predictive performance

**Figure 4:**
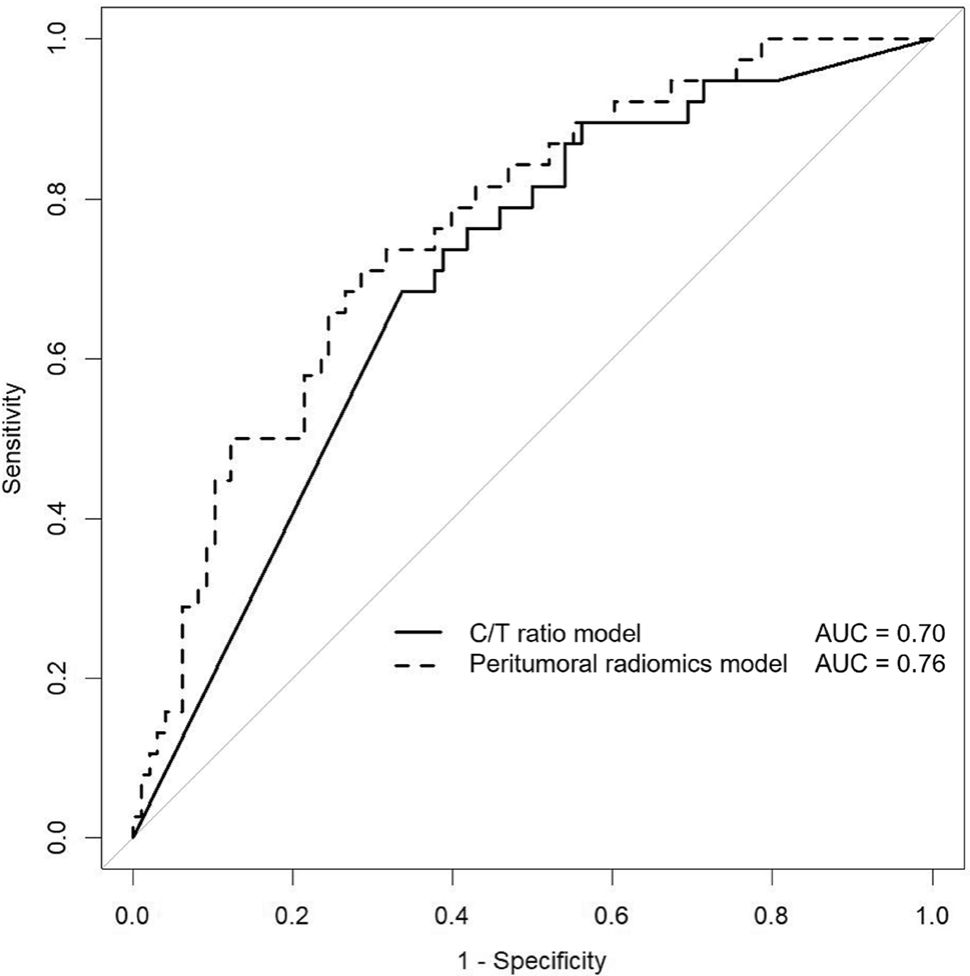
ROC curve of the C/T ratio model and the peritumoral radiomics model.

**Figure 5:**
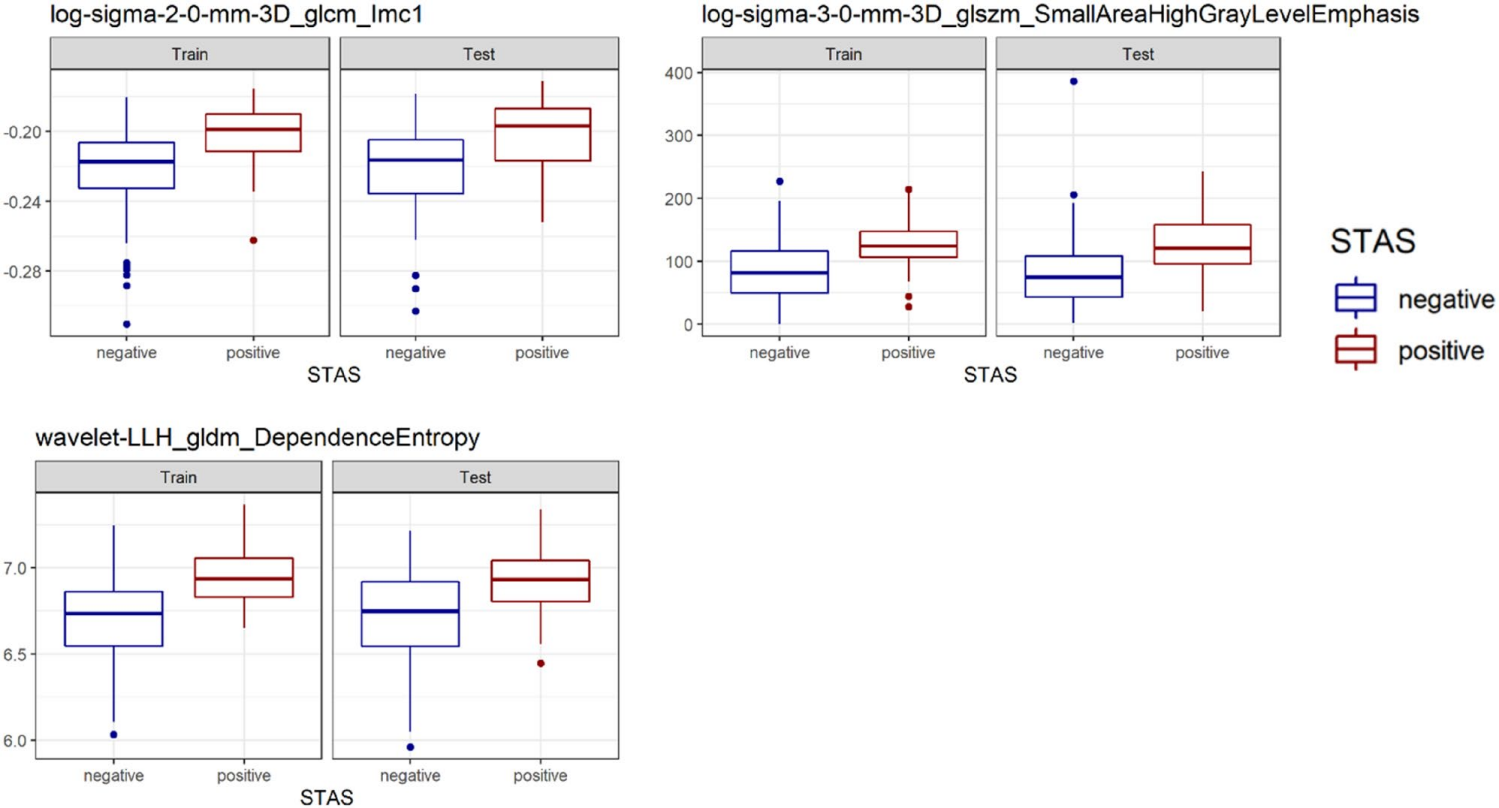
Values of features used in peritumoral radiomics model in training/test dataset.

The AUCs in the testing cohort of the peritumoral radiomics model and the C/T ratio model were 0.76 (95% CI 0.67–0.84) and 0.70 (95% CI 0.61–0.78), respectively (Fig. 4). The predictive performance of the peritumoral radiomics model was significantly higher than that of the C/T ratio model (*P* = 0.045, Table 2). The distribution of feature values used in the peritumoral radiomics model in the training and testing cohorts is shown in Fig. 5. In the testing cohort, patients at STAS-high risk according to the radiomics model were significantly associated with greater age, lobectomy, larger tumor diameter with higher C/T ratio compared with those at low risk (Table 3).

**Table 3:** Patient characteristics of the testing cohort according to the peritumoral radiomics risk model.

Characteristics	STAS high risk (n = 55)	STAS low risk (n = 81)	*P* value
Age	69 (62, 75)	66 (60, 72)	0.09
**Sex**			0.62
Male	29 (53%)	38 (47%)	
Female	26 (47%)	43 (53%)	
**Smoking status**			0.10
Current	11 (20%)	11 (14%)	
Ex	25 (45%)	27 (33%)	
Never	19 (35%)	43 (53%)	
**Location**			0.32
Left lower lobe	9 (16%)	18 (22%)	
Left upper lobe	16 (29%)	23 (28%)	
Right lower lobe	9 (16%)	13 (16%)	
Right middle lobe	6 (11%)	2 (3%)	
Right upper lobe	15 (27%)	25 (31%)	
**Surgery**			0.001
Lobectomy	44 (80%)	31 (38%)	
Partial resection	2 (4%)	11 (14%)	
Segmentectomy	9 (16%)	39 (48%)	
Diameter of consolidation (mm)	22 (17, 28)	7 (0, 13)	< 0.001
Diameter of tumor (mm)	23 (18, 31)	17 (12, 25)	< 0.001
C/T ratio	1.00 (1.00, 1.00)	0.43 (0.00, 0.70)	< 0.001
STAS	28 (51%)	10 (12%)	< 0.001

Values are presented in median (interquartile range) or number (percentage).

*C/T ratio* consolidation/tumor ratio, *STAS* spread through air spaces.

The median follow-up duration of the test group was 58 months (range 1–130 months). In the test group, 61 patients (45%) underwent limited resection (partial resection [n = 13] and segmentectomy [n = 48]), and 75 (55%) underwent lobectomy. In the limited resection group, 6 (10%) patients developed recurrence (locoregional [n = 4] and distant [n = 5]), and two (3%) died without recurrence. In contrast, 10 (13%) patients developed recurrence (locoregional [n = 4] and distant [n = 8]), and 5 (7%) died without recurrence in the lobectomy group.

**Figure 6:**
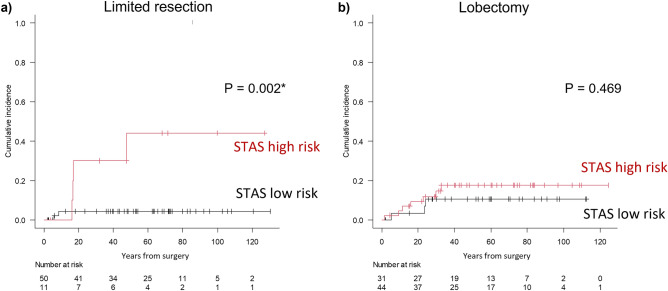
Cumulative incidence of recurrence by the cutoff value of peritumoral radiomics model in the limited resection group (**a**) and the lobectomy group (**b**).

In the testing cohort, the patients were divided into STAS high-risk and STAS low-risk groups based on the predicted probability calculated from the peritumoral radiomics model. In the limited resection group, the risk of recurrence was significantly higher in the STAS high-risk group than that in the STAS low-risk group (5-year CIR, 44% vs. 4%; *P* = 0.002) in the limited resection group (Fig. 6a). In contrast, in the lobectomy group, the risk of recurrence was not significantly different between the STAS high-risk and STAS low-risk groups (5-year CIR, 17% vs. 11%; *P* = 0.469) (Fig. 6b).

## Discussion

We demonstrated that the model using radiomics features extracted from the peritumoral ROI could significantly improve the prediction performance of STAS as compared to the conventional model using C/T ratio and could also predict the prognosis after limited surgery in lung adenocarcinoma. To the best of our knowledge, no studies so far have used STAS prediction models to predict the prognosis after limited resection. Our prediction model had the potential to diagnose patients who benefit from limited resection.

We focused on the radiomics features at the tumor margins (peritumoral radiomics) to predict the presence of STAS since STAS is a pathological finding found at the tumor margins. Toyokawa et al. reported that imaging findings of nodule margins, such as peritumoral notch and surrounding GGO, are independent predictive factors for the presence of STAS^10^. In the study by Zhuo et al., radiomics analysis was performed focusing on the outer region of the tumor, but no improvement in prediction was observed^18^. In their report, fine characteristics of the tumor margins might not have been adequately included. In our study, the marginal nature of the tumor was captured in detail by adding 5-mm margins for both inside and outside the tumor. According to Kadota et al., the median distance of STAS was 1.5 mm (range 0.2–8.5) from the tumor edge, and a 5-mm margin outside the tumor can cover about 90% of the cases where STAS was present^5^. The tumor edge defined by preoperative CT was not always accurate on CT owing to technical limitations. Chan et al. reported that CT segmentation might overestimate the edge of the tumor^21^.

Several authors reported the application of peritumoral radiomics to research in lung cancer treatment other than STAS. Wang et al. developed a nomogram for predicting lymph node metastasis after lung cancer surgery by using radiomics features extracted from tumor ROI and peritumoral ROI^22^. Akinci D'Antonoli et al. reported that the addition of radiomics features extracted from tumor ROIs and peritumoral ROIs to the TNM stage improved the prediction accuracy of recurrence rate after lung cancer surgery^23^.

As with pathological STAS, the risk of postoperative recurrence was significantly higher in the high-risk STAS group than that in the low-risk STAS group only in the limited resection group. This implies that our model could be used for additional decision-making information when selecting the surgical technique. Indeed, in the STAS low-risk group, more patients underwent limited resection compared with the STAS high. That might reflect that surgeons chose a less invasive surgery for low-risk patients according to the tumor size and C/T ratio. Masai et al. reported that the presence of STAS and tumor margins < 10 mm are significant risk factors for local recurrence in early-stage lung cancer after limited resection, and preoperative prediction of this may allow optimization of the resection margin^24^.

Our prediction model would be applied to medically inoperable patients with early-stage lung cancer, where stereotactic body radiation therapy (SBRT) is delivered. In the SBRT cases, evaluation of the presence of STAS in pathological specimens is not possible. The clinical target volume (CTV), which is the extent of tumor microinvasion, is usually defined as the same as gross tumor volume in the planning of SBRT^25,26^. An appropriate CTV margin may help local control if an accurate prediction of STAS is possible.

There are several limitations to this study. First, this was a single-center retrospective study, and prospective external model validation is needed. Second, patients who underwent limited resection included those who could not undergo lobectomy because of complications or elderly age and were subject to death due to causes other than lung cancer. The usefulness of this model in predicting the probability of STAS as a basis for clinical decision-making needs to be verified in a randomized study. Third, the radiomics features used in this model were not compared with imaging findings interpreted by radiologists; therefore, further studies are needed to identify the significance of those features. Fourth, our prediction model was solely based on imaging features because our main hypothesis was that the imaging features could reflect the information of the existence of STAS. However, clinical factors such as age, gender, and serum carcinoembryonic antigen are also reported to be predictors of STAS, and the addition of these factors may lead to a more predictive model^27^. Lastly, there is room to further reduce data sampling bias by applying sophisticated methods to building a prediction model, such as nested cross validation; however, we needed to leave a test cohort aside in advance for the following survival outcome analysis.

In conclusion, we developed a machine learning model based on peritumoral radiomics features. Its prediction performance is superior compared to that of the conventional model using the C/T ratio. Our model could further predict prognosis after limited surgery, as well as pathological STAS.

## Supplementary Information

Supplementary Information 1.

Supplementary Information 2.
