# Supplementary material for: Peritumoral radiomics features on preoperative thin-slice CT images can predict the spread through air spaces of lung adenocarcinoma
Source: Sci Rep. 2022 Jun 20;12:10323. doi: 10.1038/s41598-022-14400-w (PMC9209514; doi:10.1038/s41598-022-14400-w)
Supplement: Supplementary file 1 — Supplementary Information 1. [file 41598_2022_14400_MOESM1_ESM.docx]

**Supplementary Materials**

1. **Table E1:** Detailed CT scanning parameters.
2. **Table E2:** A full list of the radiomic features.
3. **Figure E1:** Segmentation procedures for the peritumoral ROI using the Segmentation wizard of 3D Slicer
4. ICC values for the features (intraclass_correlation.csv).

| **Table E1: Detailed CT scanning parameters** | | |
| --- | --- | --- |
|  |  | N (%) |
| CT model name | Aquillion 64 Aquillion ONE | 337 (99%) 2 (1%) |
| kVp | 120 kV | 339 (100%) |
| Slice thickness | 1 mm | 339 (100%) |
| Convolution kernel | FC11 FC13 | 55 (16%) 284(84%) |
| Pixel spacing [mm]  median (range) |  | 0.68 (0.59-0.74) |

**Table E2: Radiomic feature groups and image-preprocessing filters**

| Feature groups or filters | Parameters or descriptions | Features |
| --- | --- | --- |
| Shape (14) |  | *Elongation, Flatness, LeastAxisLength, MajorAxisLength, Maximum2DDiameterColumn, Maximum2DDiameterRow, Maximum2DDiameterSlice, Maximum3DDiameter, MeshVolume, MinorAxisLength, Sphericity, SurfaceArea, SurfaceVolumeRatio, VoxelVolume* |
| First-order (18) | VoxelArrayShift: 1000 | *10Percentile, 90Percentile, Energy, Entropy, InterquartileRange, Kurtosis, Maximum, MeanAbsoluteDeviation, Mean, Median, Minimum, Range, RobustMeanAbsoluteDeviation, RootMeanSquared, Skewness, TotalEnergy, Uniformity, Variance* |
| GLCM (22) | Distances: 1, symmetricalGLCM: True, WeightingNorm: None, Weighting: None | *Autocorrelation, ClusterProminence, ClusterShade, ClusterTendency, Contrast, Correlation, DifferenceAverage, DifferenceEntropy, DifferenceVariance, Id, Idm, Idmn, Idn, Imc1, Imc2, InverseVariance, JointAverage, JointEnergy, JointEntropy, MaximumProbability, SumEntropy, SumSquares* |
| GLRLM (16) | WeightingNorm: None, Weighting: None | *GrayLevelNonUniformity, GrayLevelNonUniformityNormalized, GrayLevelVariance, HighGrayLevelRunEmphasis, LongRunEmphasis, LongRunHighGrayLevelEmphasis, LongRunLowGrayLevelEmphasis, LowGrayLevelRunEmphasis, RunEntropy, RunLengthNonUniformity, RunLengthNonUniformityNormalized, RunPercentage, RunVariance, ShortRunEmphasis, ShortRunHighGrayLevelEmphasis, ShortRunLowGrayLevelEmphasis* |
| GLSZM (16) |  | *GrayLevelNonUniformity, GrayLevelNonUniformityNormalized, GrayLevelVariance, HighGrayLevelZoneEmphasis, LargeAreaEmphasis, LargeAreaHighGrayLevelEmphasis, LargeAreaLowGrayLevelEmphasis, LowGrayLevelZoneEmphasis, SizeZoneNonUniformity, SizeZoneNonUniformityNormalized, SmallAreaEmphasis, SmallAreaHighGrayLevelEmphasis, SmallAreaLowGrayLevelEmphasis, ZoneEntropy, ZonePercentage, ZoneVariance* |
| GLDM (14) | Distance: 1, Gldm_a: 0 | *DependenceEntropy, DependenceNonUniformity, DependenceNonUniformityNormalized, DependenceVariance, GrayLevelNonUniformity, GrayLevelVariance, HighGrayLevelEmphasis, LargeDependenceEmphasis, LargeDependenceHighGrayLevelEmphasis, LargeDependenceLowGrayLevelEmphasis, LowGrayLevelEmphasis, SmallDependenceEmphasis, SmallDependenceHighGrayLevelEmphasis, SmallDependenceLowGrayLevelEmphasis* |
| NGTDM (5) | Distance: 1 | *Busyness, Coarseness, Complexity, Contrast, Strength* |
| LoG ([525 = 105 × 5]) | Sigma: 1, 2, 3, 4, 5 | First-order and texture features with applying a Laplacian of Gaussian filter with setting sigma value σ = {1, 2, 3, 4, 5} |
| Wavelet ([840 = 105 × 8]) | Start_level: 0, Level: 1, Wavelet: coif1 | First-order and texture features with applying a wavelet filter (LLL, LLH, LHH, LHL, HLL, HLH, HHL, HHH) with a mother wavelet of Coiflet 1 |

Abbreviations: GLCM = gray level co-occurrence matrix, GLRLM = gray level run length matrix, GLSZM = gray level size zone matrix, GLDM = gray level dependence matrix, NGTDM = neighboring gray tone difference matrix, LoG = Laplacian of Gaussian filter.


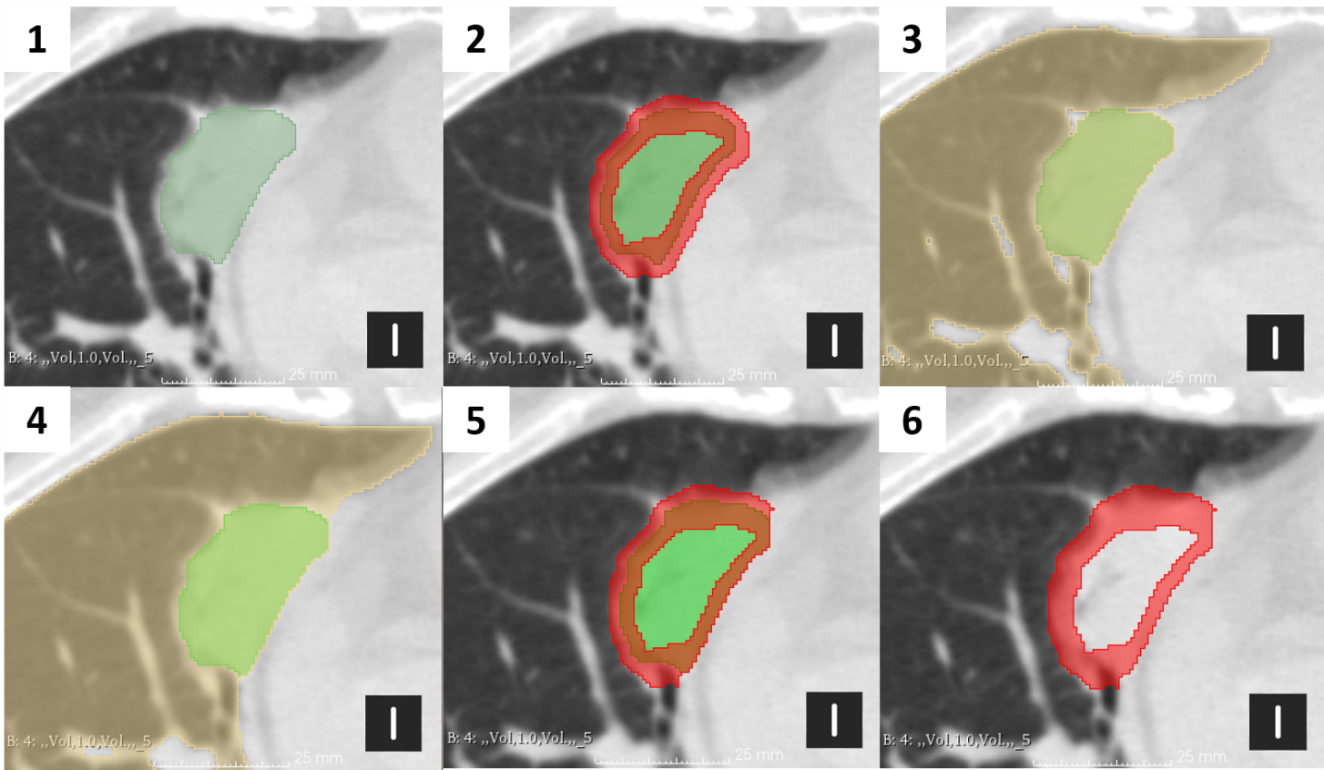


**Figure E1. Segmentation procedures for the peritumoral ROI using the Segmentation wizard of 3D Slicer**

1. Segment a tumor ROI (light green) semi-automatically using the GrowCut algorithm.

2. Expand the tumor ROI outside by 5 mm, then create a ring-like ROI (red) by removing its interior at 10 mm from the surface of the expanded ROI using the “hollow” function.

3. Create a lung ROI (yellow) by thresholding with a range of -1000 HU to -200HU.

4. Create a union set of the lung and the tumor ROIs, then fill small holes within the union set using the “fill holes smoothing” function.

5. Create a union set of the ROIs 2 and 4 in order to trim the ring-like ROI outside of the lung.

6. Finish segmentation of the peritumoral ROI (red).
